# Cross-border utilization of cancer care by patients in the US and Mexico – a survey of Mexican oncologists

**DOI:** 10.1186/s12992-023-00983-0

**Published:** 2023-10-27

**Authors:** Michael LaPelusa, Haydeé Verduzco-Aguirre, Fernando Diaz, Fernando Aldaco, Enrique Soto-Perez-de-Celis

**Affiliations:** 1 Department of Internal Medicine, Vanderbilt University Medical Center, Nashville, TN USA; 2 Department of Hemato-Oncology, Instituto Nacional de Ciencias Médicas y Nutrición Salvador Zubirán, Mexico City, Mexico; 3 Lineberger Comprehensive Cancer Center, University of North Carolina – Chapel Hill, Chapel Hill, NC United States; 4 Servicio de Oncología Medica, Centro Médico Nacional 20 de Noviembre, Mexico City, Mexico; 5 Department of Geriatrics, Instituto Nacional de Ciencias Médicas y Nutrición Salvador Zubirán, Vasco de Quiroga 15, 14080 Tlalpan, Mexico City, Mexico

**Keywords:** Emigration and immigration, Cancer, Border crossings, Mexico, Delivery of Healthcare

## Abstract

**Background:**

The US-Mexico border is the busiest in the world, with millions of people crossing it daily. However, little is known about cross-border utilization of cancer care, or about the reasons driving it. We designed a cross sectional online survey to understand the type of care patients with cancer who live in the US and Mexico seek outside their home country, the reasons why patients traveled across the border to receive care, and the barriers faced when seeking cross-border care.

**Results:**

The online survey was sent to the 248 cancer care providers working in the six Mexican border states who were registered members of the Mexican Society of Oncology. Responses were collected between September-November 2022. Sixty-six providers (response rate 26%) completed the survey. Fifty-nine (89%) reported interacting with US-based patients traveling to Mexico to receive various treatment modalities, with curative surgery (n = 38) and adjuvant chemotherapy (n = 31) being the most common. Forty-nine (74%) reported interacting with Mexico-based patients traveling to the US to receive various treatment modalities, with immunotherapy (n = 29) and curative surgery (n = 27) being the most common. The most frequently reported reason US-based patients sought care in Mexico was inadequate health insurance (n = 45). The most frequently reported reason Mexico-based patients sought care in the US was patients’ perception of superior healthcare (n = 38).

**Conclusions:**

Most Mexican oncologists working along the Mexico-US border have interacted with patients seeking or receiving binational cancer care. The type of care sought, as well as the reasons for seeking it, differ between US and Mexico-based patients. These patterns of cross-border healthcare utilization highlight unmet needs for patients with cancer in both countries and call for policy changes to improve outcomes in border regions.

**Supplementary Information:**

The online version contains supplementary material available at 10.1186/s12992-023-00983-0.

## Background

The United States (US) and Mexico share a 1,954-mile border which is crossed by over 200 million people every year, making it the busiest in the world. Estimates of how many people cross the border specifically to receive healthcare vary, but data from one analysis suggested that 4.5 million people traveled to Mexico for healthcare in the year 2007 alone [1]. In 2014, individuals traveling to Mexico to receive healthcare generated an estimated $3.1 billion US dollars in revenue [2]. Most US-based individuals who travel to Mexico to receive healthcare are US citizens who are first or second-generation Mexican immigrants, live in close geographic proximity to the border, and travel to Mexican cities near the border [3–5]. Common reasons why these individuals cross the border to get medical care include lack of health insurance making them unable to afford healthcare in the US, limited English proficiency, and a perception that care in Mexico may be more aligned with their cultural beliefs [1, 4–9]. Less is known about the reasons why patients travel from Mexico to the US for healthcare.

A major obstacle to quality cancer care in Mexico is limited availability and access to procedures, therapies, and diagnostic tests, with many patients unable to receive treatment and consequently experiencing worse outcomes [10–13]. Likewise, many underserved patients with cancer who live in the US have similar issues accessing care, primarily related to cost [14, 15].

Data pertaining to how and why patients with cancer who live in the US and Mexico travel across the border to receive care explicitly related to their cancer are limited. The objective of this work was to understand, according to Mexican oncologists practicing in border states, the reasons patients with cancer in the US and Mexico seek care outside their home country and, for those patients who do, what barriers they face.

## Methods

We administered a cross-sectional online survey to Mexican oncologists practicing in Mexican border states (Baja California, Chihuahua, Coahuila, Nuevo León, Sonora, and Tamaulipas), identified from the Mexican Society of Oncology’s (SMEO) member directory. The study is reported in accordance with the Consensus-Based Checklist for Reporting Survey Studies (CROSS) [16].

The Spanish-language survey (Appendix 1) was created by a group of Mexican and US oncologists and included 42 questions divided in three sections, dealing with (1) demographic information; (2) patients from the US seeking cancer care in Mexico; and (3) patients from Mexico seeking cancer care in the US. The survey was pretested among Spanish-speaking oncology fellows to assess the clarity of the questions. The target population were the 248 members of SMEO (surgeons, medical oncologists, radiation oncologists, gynecologist oncologists, pathologists, and pediatric oncologists) working in the six Mexican border states.

The survey was administered through REDCap between October and November 2022. An initial email advertising the survey and four weekly reminders were sent to potential respondents from the SMEO email account. Participant responses were anonymized and the IRB of *Instituto Nacional de Ciencias Médicas y Nutrición Salvador Zubirán* approved the study.

Descriptive statistics were utilized to analyze the study population and responses to the survey. Due to the survey’s characteristics, missing values were not possible. Weighing of items, use of propensity scores, or sensitivity analyses were not performed. Statistical analyses were performed utilizing StataCorp. 2021. Stata Statistical Software: Release 17. College Station, TX: StataCorp LLC.

## Results

### Respondent demographics

Sixty-six respondents began and completed the survey, corresponding to a response rate of 26.6%. Respondents identified as medical oncologists (n = 25), surgical oncologists (n = 24), gynecologic oncologists (n = 7), radiation oncologists (n = 6), pediatric oncologists (n = 2), palliative care specialists (n = 1), and hematologists (n = 1). Most respondents practiced in Baja California (n = 21) followed by Nuevo León (n = 14), Chihuahua (11), Sonora (n = 9), Tamaulipas (n = 6), and Coahuila (n = 5).

### Types of therapies, imaging, and tests patients sought outside their Home Country

Sixty (91%) respondents interacted with US-based patients who traveled to Mexico for care within the past five years, with 19 reporting interacting with ≥ 10. Fifty-nine (89%) interacted with US-based patients traveling to Mexico to receive various treatment modalities, with curative surgery (n = 38) and adjuvant chemotherapy (n = 31) being the most common. Seven (12%) interacted with US-based patients traveling to Mexico seeking non-approved therapies, including homeopathic and alternative treatments. Thirty-one (47%) interacted with US-based patients who traveled to Mexico to buy cancer medications, with oral chemotherapy (n = 23), oral hormone therapy (n = 19), and opioid analgesics (n = 17) being the most common (Table 1). Forty-six (69.7%) interacted with US-based patients traveling to Mexico for imaging, including computed tomography (CT) (n = 38), magnetic resonance imaging (MRI) (n = 33), and nuclear imaging (n = 32). Thirty-seven (56%) interacted with US-based patients traveling to Mexico for diagnostic tests such as biopsies (n = 34), bloodwork (n = 21), and tumor markers (n = 21) (Table 2).

**Table 1:** Types of therapies patients received outside of their home country according to Mexican oncologists

Therapy type	US-based patients N = 59	Mexico-based patients N = 49
Neoadjuvant chemotherapy	29 (49.2%)	23 (46.9%)
Adjuvant chemotherapy	31 (52.5%)	24 (49.0%)
Palliative chemotherapy	25 (42.4%)	24 (49.0%)
Immunotherapy	22 (37.3%)	29 (59.2%)
Hormonal therapy	20 (22.9%)	14 (28.6%)
Autologous transplant	3 (5.1%)	6 (12.2%)
Allogeneic transplant	1 (1.7%)	5 (10.2%)
CAR-T cell therapy	0 (0.0%)	5 (10.2%)
Curative surgery	38 (64.4%)	27 (55.1%)
Palliative surgery	19 (32.2%)	11 (22.4%)
Curative radiation	22 (37.3%)	16 (32.7%)
Palliative radiation	15 (25.4%)	7 (14.3%)
Non-approved therapies	7 (11.9%)	0 (0.0%)
Symptom management	16 (27.1%)	9 (18.4%)

**Table 2:** Types of diagnostic imaging and tests patients receive outside of their home country according to Mexican oncologists

		US-based patients N = 46	Mexico-based patients N = 26
**Diagnostic Imaging**	Computed tomography	38 (82.6%)	10 (38.5%)
	Magnetic resonance imaging	33 (71.1%)	10 (38.5%)
	Nuclear	32 (69.6%)	21 (80.0%)
		**US-based patients** N = 37	**Mexico-based patients** N = 23
**Diagnostic Testing**	Biopsy	34 (91.9%)	8 (36.4%)
	Basic bloodwork	21 (56.8%)	4 (18.2%)
	Blood-based tumor markers	21 (56.8%)	5 (22.7%)
	Next-generation sequencing	5 (13.9%)	20 (90.9%)

Forty-nine (74%) respondents interacted with Mexico-based patients traveling to the US for treatment, with immunotherapy (n = 29) and curative surgery (n = 27) being the most common. Twenty (30%) interacted with Mexico-based patients traveling to the US to buy cancer medications, with oral chemotherapy (n = 12) and hormonal therapy (n = 9) being the most common. Five interacted with patients seeking CAR-T therapy in the US, which is not currently available in Mexico (Table 1). Twenty-six (39%) interacted with Mexico-based patients traveling to the US for imaging, including nuclear imaging (n = 21), CT (n = 10), and MRI (n = 10). Twenty-three (35%) interacted with Mexico-based patients traveling to the US to for diagnostic tests, the most common being next-generation sequencing (NGS) (n = 20) (Table 2).

### Reasons patients pursued Cancer Care outside thier Home Country

**Fig. 1:**
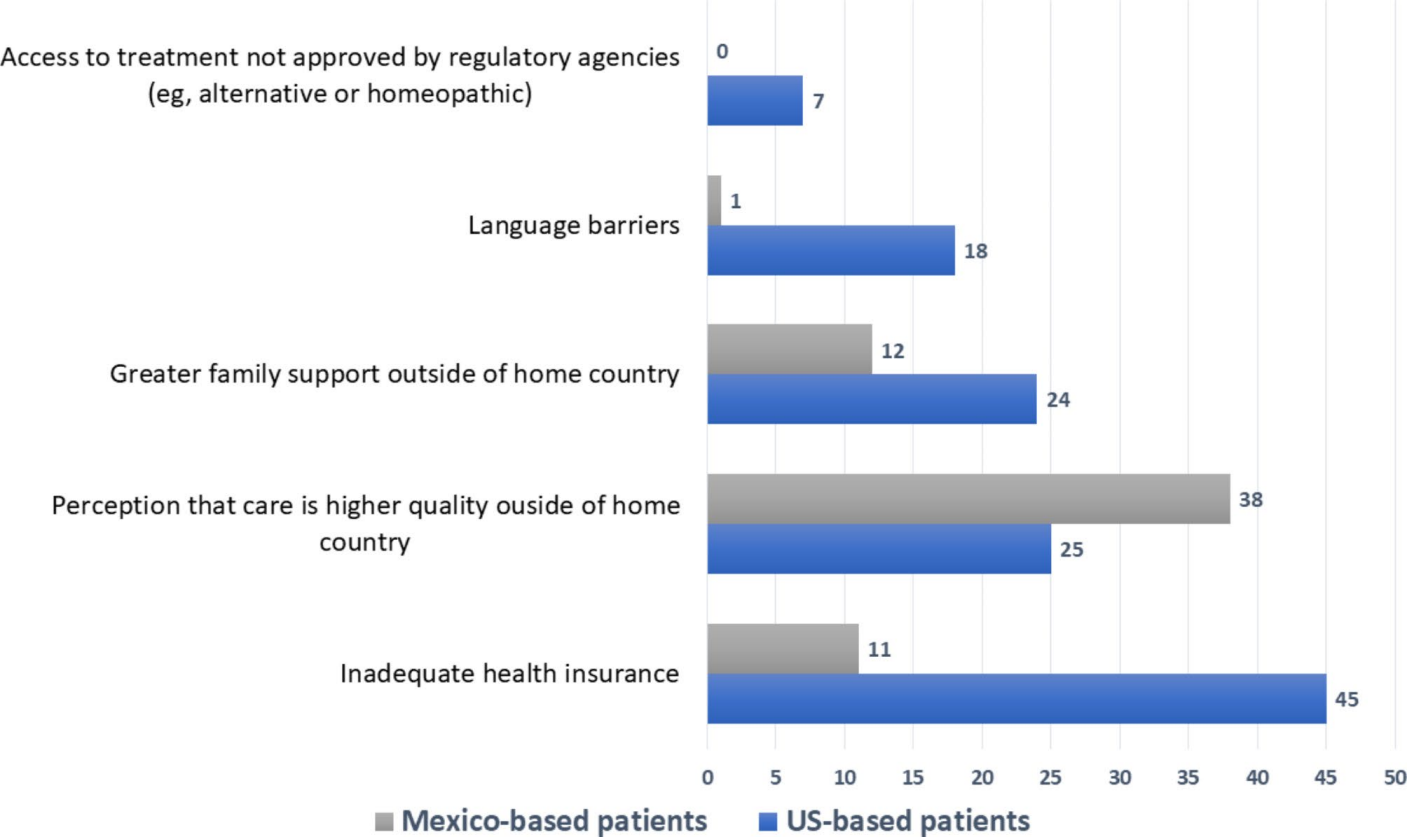
Reasons patients pursued cancer care outside of their home country according to Mexican oncologists

The main reason US-based patients sought care in Mexico was inadequate health insurance (n = 45) (Fig. 1). Out of pocket expenses were reported as the main reason why US-based patients traveled to Mexico to buy medications (n = 26), undergo imaging studies (n = 37) and get laboratory tests (n = 30) (Table 3).

**Table 3:** Reasons patients pursued treatment, diagnostic imaging, and diagnostic testing outside of their home country according to Mexican oncologists

	US-based patients			Mexico-based patients		
	Buy Medications N = 31	Undergo Imaging N = 45	Receive Tests N = 37	Buy Medications N = 20	Undergo Imaging N = 26	Receive Tests N = 22
Inability to afford out-of-pocket expenses	26 (83.9%)	37 (82.2%)	30 (81.1%)	11 (55.0%)	5 (19.2%)	5 (22.7%)
Inability to afford deductible cost or co-payment	21 (67.7%)	29 (64.4%)	22 (59.5%)	2 (10.0%)	7 (26.0%)	7 (31.8%)
Perception that medical care outside of home country is of higher quality	2 (6.5%)	5 (11.1%)	2 (5.4%)	7 (35.0%)	16 (61.5%)	14 (63.6%)
Lack of availability in home country	1 (3.2%)	0 (0.0%)	0 (0.0%)	15 (75.0%)	13 (50.0%)	9 (40.9%)

The main reason Mexico-based patients sought care in the US was patients’ perception of superior healthcare (n = 38) (Fig. 1). Lack of availability was reported as the main reason why Mexico-based patients traveled to the US to buy medications (n = 15) and to undergo imaging studies (n = 15), while the most common reason for traveling to get laboratory tests was a perception of higher quality testing in the US (n = 14) (Table 3).

### Barriers to receiving Care outside of the patients’ home country

**Fig. 2:**
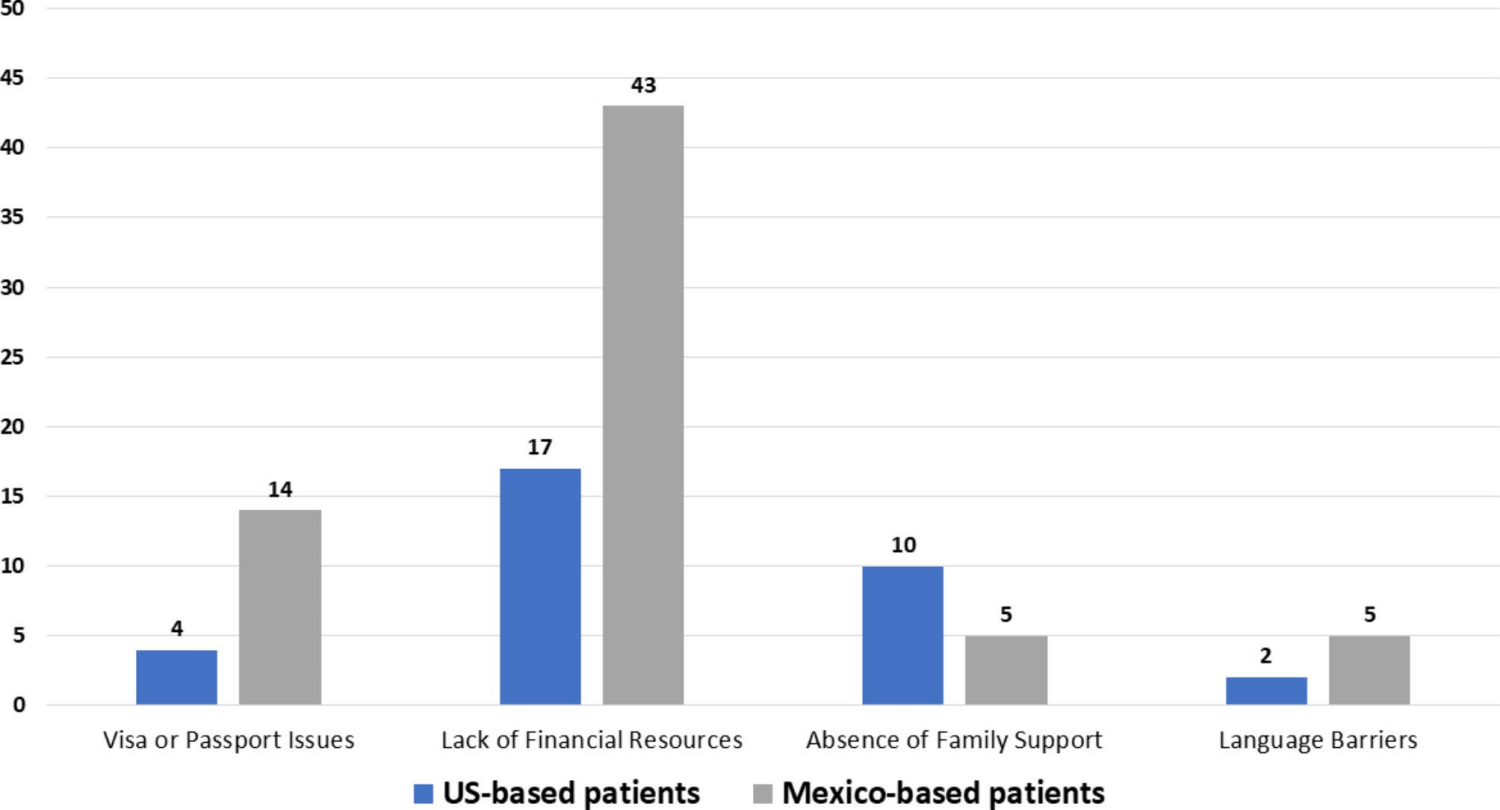
Reasons patients could not travel outside of their home country to receive cancer care according to Mexican oncologists

Twenty-nine (44%) respondents interacted with US-based patients who wanted to receive care in Mexico but could not, mostly due to lack of financial resources (n = 17). Forty-three (65%) interacted with Mexico-based patients who wanted to receive care in the US but could not, which was also mostly due to limited financial resources (n = 43) (Fig. 2).

## Discussion

This study represents a comprehensive record of cancer care utilization across the US-Mexico border, with information obtained from cancer care providers working in Mexican border states. Our results show that the scope of cancer care utilization outside of patients’ home countries is considerable, and mostly driven by financial issues, availability of tests and medications, and a perception of superior care abroad [4, 7, 8, 17]. Our findings are consistent with prior studies of bidirectional healthcare utilization in the US and Mexico, particularly concerning the limited accessibility of certain therapies and recently developed diagnostic tests, such as CAR-T or NGS.

Although efforts have been made to provide universal access for cancer care in Mexico, many patients are still required to pay for medications and other healthcare expenses, which may sometimes be unaffordable. In an analysis of National Health and Nutrition Survey data, over 17% of beneficiaries of public healthcare systems could not obtain prescribed medications [18]. Since 2020, the National Fund for Wellbeing (FONSABI) has overseen financing catastrophic health expenses, such as cancer care, although the supply of medications and access to many interventions has been inconsistent since its inception [19, 20]. The limited availability of cancer drugs in Mexico is highlighted by the fact that the average availability of essential cancer medicines (as defined by the World Health Organization) covered by Mexican public health insurance is of approximately 60% [21, 22].

US-based patients who traveled to Mexico were more likely to be seeking chemotherapy, surgery, and radiation, which may be due to the lower cost of these treatments in Mexico. In contrast, Mexico-based patients traveled to the US to obtain therapies which are either unavailable or not covered in Mexico such as immunotherapy, stem cell transplantation, and cellular therapy [23, 24]. Interestingly, US-based patients traveled to Mexico often to undergo imaging and other diagnostic tests, which may be due to their affordability and accessibility, particularly for patients with limited health insurance. Mexico-based patients traveled to the US to receive more novel tests, such as NGS or nuclear medicine, which may be due to limited availability/coverage of such testing in Mexico [25].

The most common reason reported for US-based patients traveling to Mexico was inadequate health insurance coverage. Disruptions in health insurance coverage are common among patients undergoing cancer treatment in the US and are associated with worse survival [26]. Delays and denial of medical care due to health insurance review, also known as prior authorization, have also resulted in a significant adverse impact on treatment initiation, receipt of diagnostic imaging, and out-of-pocket expenses in patients with cancer [27]. These issues likely lead some patients in the US to seek care in Mexico. Other reasons why US-based patients traveled to Mexico included access to alternative therapies, language barriers in the US, and availability of a more robust social support system in Mexico. These findings correspond to previous reports highlighting the use of alternative medicine as a major driver for individuals in the US seeking care in Mexico [28]. Conversely, Mexico-based patients traveled to the US primarily due to patients’ perception that cancer care was of higher quality in the US and that medications, imaging studies, and other diagnostic tests were unavailable in Mexico. Overall, fewer respondents reported interacting with Mexico-based patients who traveled to the US to buy medications, undergo imaging, and receive diagnostic tests than the number of respondents who reported interacting with US-based patients who traveled to Mexico for these services, which most likely is due to financial issues.

Lack of financial resources was the most common limiting factor among patients who wanted to go across the border to receive care but could not. The cost associated with traveling, lodging, and reduced income related to loss of employment from taking days off from work have been shown to impose significant financial hardship on patients with cancer in both the US and Mexico [29, 30]. A significant proportion of Mexico-based patients were unable to travel to the US to receive cancer care because of passport/visa issues and language barriers, while many US-based patients were unable to travel to Mexico to receive cancer care because of the absence of a support system in Mexico.

Our results may have implications for binational and cross-border policy. In Mexico, patients with cancer can receive care from institutions in the private or public sector. Patients who receive care at private pharmacies and health facilities typically contribute financially to private insurance companies and pay out-of-pocket expenses [31, 32]. Patients treated at institutions in the public sector usually have social health insurance, which unfortunately may be limited due to medication shortages or access issues [31, 33]. In the US, patients with cancer typically receive care from National Cancer Institute (NCI) comprehensive cancer centers, NCI-designated cancer centers, or community cancer practices. Patients with cancer who lack health insurance in the US are more likely to be diagnosed with advanced-stage cancer at diagnosis and have worse survival after diagnosis [34–36]. People living in the US along the US-Mexico border have lower health insurance rates [37], with some reports estimating the percentage of uninsured individuals in border counties at nearly 50% [38, 39]. One proposed solution to providing care to undocumented immigrants, as well as to address the liberal utilization of healthcare in both the US and Mexico by people who lack health insurance, is a binational health insurance plan. Two such programs were “*Salud Migrante*” for uninsured Mexican immigrants and “Medicare in Mexico” for older Americans [40]. Theoretically, these programs would allow undocumented immigrants to travel to Mexico to receive care while their legal status is in flux and enable Americans eligible for Medicare to travel to Mexico for components of their healthcare, respectively. Ultimately, these programs were hampered by legal and regulatory challenges and had to be discontinued.

The impact of bidirectional healthcare utilization by patients in the US and Mexico is clear in Mexican border cities, where more private (compared to public) primary care providers exist to serve patients from the US seeking care in Mexico [41]. At a patient level, bidirectional healthcare utilization may also have a significant impact. Patients who travel outside their home country to receive care often receive concurrent treatment by providers in two sites, leading to duplication of diagnostic tests and treatments [42]. This phenomenon could theoretically result in increased healthcare costs and raises a multitude of patient safety concerns. Further, the quality of healthcare provided along the Mexican border, particularly concerning elective procedures, wellness services, and fertility expertise, has been called into question [2, 43]. Risks such as lack of appropriate longitudinal care, the acquisition of multi-drug resistant organisms during surgical procedures, and lack of standardized quality control measures have led to instances of significant morbidity and mortality in patients traveling to Mexico for these services [2].

A highly complex issue affecting cross-border care is the existence of legal barriers related to immigration, as highlighted by our study findings showing a significant proportion of patients saw passport/visa issues as a barrier for getting care. Over 20 million noncitizens are currently living in the US, of which almost half are uninsured [44]. In the US, undocumented immigrants are ineligible to obtain federal health insurance offered by the Federal Health Insurance Market Place as a provision of the Affordable Care Act [45, 46]. Those seeking asylum in the US are eligible for Medicaid or other forms of US-based public insurance, while qualified noncitizens can buy insurance coverage in the marketplace during their first five years in the country, becoming eligible for Medicaid after living in the US for five years. Undocumented immigrants do not qualify for these insurance plans and may be unlikely to travel to Mexico to receive healthcare while their legal status is uncertain [1, 44, 46]. The Emergency Medical Treatment and Labor Act requires the provision of healthcare to patients in the emergency department regardless of a patient’s ability to pay. However, these services are only funded for Medicaid-eligible patients through the federally funded Emergency Medicaid program, and some border states, such as Texas, have policies restricting Medicaid eligibility [47]. On the other hand, California plans to expand Medicaid coverage to all residents older than 26 with a certain income by 2024. Notably, costs associated with providing care to US-based noncitizens are lower than those associated with providing care to citizens [48].

Tracking cancer incidence and mortality in the US-Mexico border region is challenging, partly because patients travel across the border to receive care. Some have proposed that lower cancer-related mortality rates among the Hispanic population in the US are due to the so-called “salmon-bias” effect, which purports patients return to their country of origin when they receive a terminal diagnosis [49–52]. As a result, these patients’ deaths may not be captured in national registries, resulting in an inaccurate registered mortality rate.

Our study has limitations. Firstly, the information collected from respondents was based on recall rather than prospectively collected data, which highlights the potential for recall bias on the part of respondents. This highlights a potential opportunity to gather pertinent information on patients with cancer who travel internationally to receive care. Secondly, oncologists’ perspectives on why patients traveled outside their home country to receive cancer care may have been assumed. It is plausible that asking patients themselves may have yielded different results. Further, the relatively small sample size of respondents may not accurately capture the entirety of perspectives of practicing oncologists in Mexican border states. Lastly, the descriptive nature of our study make drawing statistically significant differences between the reasons US-based and Mexico-based patients traveled to receive care and the type of therapies and studies they received and underwent difficult. However, since SMEO is the largest organization in Mexico, we believe we were able to target most oncology professionals in the border area, and the proportion of responses is standard for an online survey.

## Conclusions

According to Mexican oncologists, US-based patients travel to Mexico to receive cancer care primarily due to inadequate health insurance and excessive out of pocket expenses in the US. In contrast, Mexico-based patients travel to the US to receive cancer care due to patients’ perception that cancer care in the US is of higher quality and because diagnostic or therapeutic components of their care are unavailable in Mexico. Further, the type of care and therapies patients seek outside their home country differs, with US-based patients traveling to Mexico primarily to undergo imaging studies, biopsies, surgery, and chemotherapy; and Mexico-based patients traveled to the US to receive advanced laboratory tests (such as NGS), immunotherapy, and surgery.

These patterns of cross-border healthcare utilization highlight unmet needs for patients with cancer in the US and Mexico and are consistent with those identified by others who seek to determine why patients with cancer travel outside of their home country for care and what challenges these patients face [53]. In the US, considering the preferences and healthcare-related issues afflicting patients with cancer is essential when formulating and adopting policies related to improving access to culturally competent care. In Mexico, improving access, both in terms of affordability and availability within the Mexican healthcare system, must be addressed to provide higher-quality care.

### Electronic supplementary material

Below is the link to the electronic supplementary material.

Supplementary Material 1

## Data Availability

Data and materials are available upon request from the corresponding author.

## Abbreviations

US: United States
SMEO: Mexican Society of Oncology
CROSS: Consensus-Based Checklist for Reporting Survey Studies
CT: Computed tomography
MRI: Magnetic resonance imaging
NGS: Next-generation sequencing
CAR-T:FONSABI: National Fund for Wellbeing
NCI: National Cancer Institute
