# Supplementary material for: Cross-border utilization of cancer care by patients in the US and Mexico – a survey of Mexican oncologists
Source: Global Health. 2023 Oct 27;19:78. doi: 10.1186/s12992-023-00983-0 (PMC10612194; doi:10.1186/s12992-023-00983-0)
Supplement: Supplementary file 1 — Supplementary Material 1 [file 12992_2023_983_MOESM1_ESM.pdf]

Encuesta de utilización de atención hematológica y oncológica de Estados Unidos y México

Esta encuesta tiene el objetivo de describir las características de la atención de pacientes con cáncer residentes en ambos lados de la frontera norte de México.

El protocolo de investigación asociado ha sido aprobado por el comité de ética en investigación del Instituto Nacional de Ciencias Médicas y Nutrición Salvador Zubirán (clave HEM-4323-22-23-1).

La encuesta está dirigida a especialistas en oncología, hematología, cirugía oncológica, ginecología oncológica, radiooncología, oncología pediátrica o cuidados paliativos que laboran en alguno de los siguientes estados de la frontera norte de México: Baja California, Chihuahua, Coahuila, Nuevo León, Sonora o Tamaulipas. Contestarla le tomará alrededor de 5 minutos.

La información que usted brinde será confidencial, y los participantes podrán ingresar a un sorteo para ganar una tablet Android.

Si usted desea participar y otorga su consentimiento para el uso anónimo de sus respuestas, marque la siguiente casilla para continuar.	◯ Acepto participar dentro del protocolo y otorgo mi consentimiento para el uso anónimo de mis respuestas.
¿Cuál es su especialidad?	◯ Hemato-oncología<br>◯ Oncología médica (adultos)<br>◯ Oncología pediátrica<br>◯ Hematología (adultos)<br>◯ Hematología pediátrica<br>◯ Cirugía oncológica<br>◯ Ginecología oncológica<br>◯ Radio-oncología<br>◯ Cuidados paliativos
¿Por cuántos años ha ejercido su especialidad?	◯ < 5 años<br>◯ 6-10 años<br>◯ 11-15 años<br>◯ 16-20 años<br>◯ >20 años
¿En qué estado se encuentra su práctica clínica?	◯ Baja California<br>◯ Sonora<br>◯ Chihuahua<br>◯ Coahuila<br>◯ Nuevo León<br>◯ Tamaulipas<br>◯ Un estado diferente
¿Cómo clasificaría su sitio de práctica clínica?	◯ Institución pública<br>◯ Práctica privada<br>◯ Ambas, pública y privada
¿En qué institución o grupo se encuentra su práctica clínica?	______________________________

Las siguientes preguntas se refieren a pacientes que viven en los Estados Unidos (y que han sido vistos al menos una vez por un oncólogo en EUA) y que buscan una segunda opinión en México (y/o transfieren su atención a un especialista en México).

En los últimos 5 años, ¿ha atendido pacientes que hayan viajado a México para una segunda opinión?	◯ Sí<br>◯ No
En los últimos 5 años, ¿cuántos pacientes que hayan viajado a México para una segunda opinión ha atendido por año?	◯ 1-10<br>◯ 11-20<br>◯ 21-30<br>◯ 31-40<br>◯ 41-50<br>◯ Más de 50
¿Qué nacionalidad tienen los pacientes residentes en EUA que ha atendido en los últimos 5 años?	☐ Mexicana<br>☐ Americana<br>☐ Doble nacionalidad (mexicana y americana)<br>☐ Otra nacionalidad
En su opinión, ¿cuáles son las principales razones por las que los pacientes eligen buscar una segunda opinión en México? Seleccione todas las que apliquen.	☐ Falta de recursos económicos<br>☐ Falta de seguro médico / seguro médico inadecuado<br>☐ Percepción de que la atención médica es de mayor calidad en México<br>☐ Mayor apoyo familiar en México<br>☐ Preocupación acerca de su estatus migratorio si reciben tratamiento en EUA<br>☐ Barreras de idioma en EUA<br>☐ Acceso a tratamientos no aprobados por la FDA (p. ej. suplementos, tratamientos homeopáticos u otros tratamientos alternativos)<br>☐ Cuidados de hospicio
¿Qué tratamientos han decidido recibir en México estos pacientes? Seleccione todas las que apliquen.	☐ Terapia sistémica - quimioterapia neoadyuvante<br>☐ Terapia sistémica - quimioterapia adyuvante<br>☐ Terapia sistémica - quimioterapia paliativa (p. ej. para enfermedad metastásica)<br>☐ Terapia sistémica - inmunoterapia<br>☐ Terapia sistémica - tratamiento hormonal<br>☐ Trasplante autólogo de médula ósea/células progenitoras hematopoyéticas<br>☐ Trasplante alogénico de médula ósea/células progenitoras hematopoyéticas<br>☐ Cirugía - con intención paliativa (p. ej. por obstrucción maligna)<br>☐ Cirugía - con intención curativa<br>☐ Radioterapia - con intención paliativa (p. ej. por obstrucción maligna)<br>☐ Radioterapia - con intención curativa<br>☐ Cuidados paliativos<br>☐ Manejo del dolor<br>☐ Tratamientos no aprobados por la FDA (p. ej. suplementos, tratamientos homeopáticos u otros tratamientos alternativos)
¿Ha atendido pacientes que hayan expresado su deseo de recibir atención médica en México pero que no les haya sido posible?	◯ Sí<br>◯ No

Si no, ¿cuál fue la razón por la que esos pacientes no pudieron recibir atención en México? Seleccione todas las que apliquen.	☐ Falta de visa o pasaporte<br>☐ Falta de recursos económicos<br>☐ Falta de apoyo familiar<br>☐ No hablar español
¿Ha atendido pacientes que viajen a México para comprar medicamentos relacionados a su diagnóstico de cáncer?	◯ Sí<br>◯ No
¿Qué medicamentos suelen comprar los pacientes en México? Seleccione todos los que apliquen.	☐ Quimioterapia oral<br>☐ Tratamientos hormonales orales<br>☐ Antieméticos<br>☐ Analgésicos opioides<br>☐ Analgésicos no opioides<br>☐ Laxantes<br>☐ Tratamientos no aprobados por la FDA (p. ej. suplementos, tratamientos homeopáticos u otros tratamientos alternativos)
En su opinión, ¿cuáles son las principales razones por las que los pacientes viajan a México para comprar medicamentos o tratamientos? Seleccione todas las que apliquen.	☐ El medicamento no está cubierto por su seguro, o tiene alto deducible o co-pago<br>☐ No cuenta con seguro médico, o el medicamento es muy caro para pagar de su bolsillo<br>☐ El medicamento no se encuentra disponible en EUA<br>☐ Percepción de que los medicamentos o tratamientos son de mayor calidad en México
¿Ha atendido pacientes que viajen a México para realizarse estudios de imagen?	◯ Sí<br>◯ No
¿Qué estudios de imagen suelen realizarse los pacientes en México? Seleccione todos los que apliquen.	☐ Tomografía computarizada<br>☐ Resonancia magnética<br>☐ Estudios de medicina nuclear (p. ej. PET, gammagrama óseo, gammagrama tiroideo)<br>☐ Otros estudios de imagen
En su opinión, ¿cuáles son las principales razones por las que los pacientes viajan a México para realizarse estudios de imagen? Seleccione todas las que apliquen.	☐ El estudio no está cubierto por su seguro, o tiene alto deducible o co-pago<br>☐ No cuenta con seguro médico, o el estudio es muy caro para pagar de su bolsillo<br>☐ El estudio no se encuentra disponible en EUA<br>☐ Percepción de que los estudios de imagen son de mayor calidad en México
¿Ha atendido pacientes que viajen a México para un procedimiento diagnóstico o prueba de laboratorio?	◯ Sí<br>◯ No
¿Qué procedimientos diagnósticos o pruebas de laboratorio suelen realizarse los pacientes en México? Seleccione todos los que apliquen.	☐ Biopsia<br>☐ Biometría hemática, química sanguínea<br>☐ ACE, CA19-9 u otro marcador tumoral<br>☐ Estudios avanzados de laboratorio (p. ej. secuenciación de próxima generación [NGS])

En su opinión, ¿cuáles son las principales razones por las que los pacientes viajan a México para realizarse procedimientos diagnósticos o pruebas de laboratorio? Seleccione todas las que apliquen.

- ☐ El procedimiento o prueba no está cubierto por su seguro, o tiene alto deducible o co-pago
- ☐ No cuenta con seguro médico, o el procedimiento o prueba es muy caro para pagar de su bolsillo
- ☐ El procedimiento o prueba no se encuentra disponible en EUA
- ☐ Percepción de que los procedimientos o pruebas son de mayor calidad en México

Las siguientes preguntas se refieren a TODOS los pacientes que usted ha atendido que son de origen estadounidense.

¿Ha atendido pacientes que ya no eran candidatos a quimioterapia o que pasaron a cuidados de hospicio y que expresaron su deseo de regresar a su país natal (EUA) por el resto de su vida?

- ◯ Sí
- ◯ No

En los últimos 5 años, ¿aproximadamente cuántos pacientes ha atendido que hayan dejado México para ir a EUA por esta razón?

____

En su opinión, ¿cuáles son las principales razones por las que los pacientes se regresan a EUA cuando no tienen más opciones de tratamiento oncológico? Seleccione todas las que apliquen.

- ☐ Los cuidados de hospicio no están cubiertos por su seguro o tienen alto deducible o co-pago.
- ☐ No cuenta con seguro médico, o los cuidados de hospicio son muy caros para pagar de su bolsillo
- ☐ Para estar con su familia y/o seres queridos en EUA

Las siguientes preguntas se refieren a pacientes que viven en México (y que han sido vistos al menos una vez por un oncólogo en México) y que buscan una segunda opinión en EUA (y/o transfieren su atención a un especialista en EUA).

En los últimos 5 años, ¿ha atendido pacientes que hayan viajado a EUA para una segunda opinión?	◯ Sí<br>◯ No
En los últimos 5 años, ¿cuántos pacientes que hayan viajado a EUA para una segunda opinión ha atendido?	______________________________
En su opinión, ¿cuáles son las principales razones por las que los pacientes eligen buscar una segunda opinión en EUA? Seleccione todas las que apliquen.	☐ Falta de seguro médico / seguro médico inadecuado<br>☐ Percepción de que la atención médica es de mayor calidad en EUA<br>☐ Mayor apoyo familiar en EUA<br>☐ Barreras de idioma en México<br>☐ Cuidados de hospicio
¿Qué tratamientos han decidido recibir en EUA estos pacientes? Seleccione todas las que apliquen.	☐ Terapia sistémica - quimioterapia neoadyuvante<br>☐ Terapia sistémica - quimioterapia adyuvante<br>☐ Terapia sistémica - quimioterapia paliativa (p. ej. para enfermedad metastásica)<br>☐ Terapia sistémica - inmunoterapia<br>☐ Terapia sistémica - tratamiento hormonal<br>☐ Trasplante autólogo de médula ósea/células progenitoras hematopoyéticas<br>☐ Trasplante alogénico de médula ósea/células progenitoras hematopoyéticas<br>☐ CAR-T<br>☐ Cirugía - con intención paliativa (p. ej. por obstrucción maligna)<br>☐ Cirugía - con intención curativa<br>☐ Radioterapia - con intención paliativa (p. ej. por obstrucción maligna)<br>☐ Radioterapia - con intención curativa<br>☐ Cuidados paliativos<br>☐ Manejo del dolor
¿Ha atendido pacientes que hayan expresado su deseo de recibir atención médica en EUA pero que no les haya sido posible?	◯ Sí<br>◯ No
¿Cuál fue la razón por la que esos pacientes no pudieron recibir atención en EUA? Seleccione todas las que apliquen.	☐ Falta de visa o pasaporte<br>☐ Falta de recursos económicos<br>☐ Falta de apoyo familiar<br>☐ No hablar inglés
¿Ha atendido pacientes que viajen a EUA para comprar medicamentos relacionados a su diagnóstico de cáncer?	◯ Sí<br>◯ No
¿Qué medicamentos suelen comprar los pacientes en EUA? Seleccione todos los que apliquen.	☐ Quimioterapia oral<br>☐ Tratamientos hormonales orales<br>☐ Antieméticos<br>☐ Analgésicos opioides<br>☐ Analgésicos no opioides<br>☐ Laxantes

En su opinión, ¿cuáles son las principales razones por las que los pacientes viajan a EUA para comprar medicamentos o tratamientos? Seleccione todas las que apliquen.	☐ El medicamento no está cubierto por su seguro, o tiene alto deducible o co-pago<br>☐ No cuenta con seguro médico, o el medicamento es muy caro para pagar de su bolsillo<br>☐ El medicamento no se encuentra disponible en México<br>☐ Percepción de que los medicamentos o tratamientos son de mayor calidad en EUA
¿Ha atendido pacientes que viajen a EUA para realizarse estudios de imagen?	◯ Sí<br>◯ No
¿Qué estudios de imagen suelen realizarse los pacientes en EUA? Seleccione todos los que apliquen.	☐ Tomografía computarizada<br>☐ Resonancia magnética<br>☐ Estudios de medicina nuclear (p. ej. PET, gammagrama óseo, gammagrama tiroideo)<br>☐ Otros estudios de imagen
En su opinión, ¿cuáles son las principales razones por las que los pacientes viajan a EUA para realizarse estudios de imagen? Seleccione todas las que apliquen.	☐ El estudio no está cubierto por su seguro, o tiene alto deducible o co-pago<br>☐ No cuenta con seguro médico, o el estudio es muy caro para pagar de su bolsillo<br>☐ El estudio no se encuentra disponible en México<br>☐ Percepción de que los estudios de imagen son de mayor calidad en EUA
¿Ha atendido pacientes que viajen a EUA para un procedimiento diagnóstico o prueba de laboratorio?	◯ Sí<br>◯ No
¿Qué procedimientos diagnósticos o pruebas de laboratorio suelen realizarse los pacientes en EUA? Seleccione todos los que apliquen.	☐ Biopsia<br>☐ Biometría hemática, química sanguínea<br>☐ ACE, CA19-9 u otro marcador tumoral<br>☐ Estudios avanzados de laboratorio (p. ej. secuenciación de próxima generación [NGS])
En su opinión, ¿cuáles son las principales razones por las que los pacientes viajan a EUA para realizarse procedimientos diagnósticos o pruebas de laboratorio? Seleccione todas las que apliquen.	☐ El procedimiento o prueba no está cubierto por su seguro, o tiene alto deducible o co-pago<br>☐ No cuenta con seguro médico, o el procedimiento o prueba es muy caro para pagar de su bolsillo<br>☐ El procedimiento o prueba no se encuentra disponible en México<br>☐ Percepción de que los procedimientos o pruebas son de mayor calidad en EUA
Si desea participar en el sorteo de una tarjeta de regalo de Amazon, por favor ingrese un correo electrónico de contacto. Le recordamos que los datos proporcionados y sus respuestas serán confidenciales.	______________________________
Por favor ingrese nuevamente su correo electrónico.	______________________________
